# PPAR Could Contribute to the Pathogenesis of Hepatocellular Carcinoma

**DOI:** 10.1155/2012/574180

**Published:** 2012-12-16

**Authors:** Osamu Kimura, Yasuteru Kondo, Tooru Shimosegawa

**Affiliations:** Division of Gastroenterology, Tohoku University Hospital, 1-1 Seiryo-Machi, Aoba-ku, Sendai City, Miyagi 980-8574, Japan

## Abstract

Viral hepatitis with hepatitis C virus or hepatitis B virus and chronic liver disease such as alcoholic or nonalcoholic steatohepatitis are critical factors in the development of hepatocellular carcinoma (HCC). Furthermore, diabetes is known as an independent risk factor for HCC. Peroxisome proliferator-activated receptor (PPAR) is known to have an important role in fatty liver, and the mechanism of carcinogenesis has been clarified. PPAR controls ligand-dependent transcription, and three subtypes (**α**, **δ**, and **γ**) in humans are known. PPARs could contribute to the mechanisms of cell cycling, anti-inflammatory responses, and apoptosis. Therefore, to clarify the pathogenesis of HCC, we should examine PPAR signaling. In this paper, we have summarized the relevance of PPARs to the pathogenesis of HCC and cancer stem cells and possible therapeutic options through modifying PPAR signaling.

## 1. Introduction

Worldwide, the mortality from hepatocellular carcinoma represents one-third of all cancer deaths with more than 1 million a year [1]. In the early stages of hepatocellular carcinoma, when patients maintain a hepatic functional reserve, local treatment such as hepatic resection or radio-frequency ablation is relatively effective [2]. However, many patients die from repeated recurrence. Hepatic arterial infusion chemotherapy (HAIC) [3, 4] for advanced hepatocellular carcinoma is only sometimes effective. Also, sorafenib (VEGF-2/PDGFR-beta inhibitor) [5], which has come in to use recently, has not shown satisfactory results [6, 7]. Therefore, finding a new therapeutic target molecule has become very important.

Viral hepatitis with HCV or HBV and chronic liver disease such as alcohol or nonalcoholic steatohepatitis (NASH) [8] are critical factors in the development of hepatocellular carcinoma. Furthermore, diabetes is known as an independent risk factor for hepatocellular carcinoma [9, 10]. In addition, the hepatitis C is known to become fatty liver at a high rate [11, 12]. Peroxisome proliferator-activated receptor (PPAR) [13, 14] plays an important role in fatty liver, and its involvement in carcinogenesis has been clarified. PPAR controls ligand-dependent transcription, and three subtypes (*α*, *δ*, and *γ*) in humans are known. PPAR*α* [14] is present in liver, kidney, heart, and small intestine and has an important role in the regulation of lipid metabolism [15]. PPAR*γ* is expressed in adipose tissue and macrophages. It is involved in adipose cell differentiation and lipid uptake and has anti-inflammatory effects. In addition, PPAR*γ* expression is induced in the liver in a hypernutrition state such as fatty liver. PPAR*δ* is expressed universally. It is involved in fatty acid metabolism and the induction of energy in skeletal muscle and adipose tissue.

 PPARs have roles regulating the cell cycle and metabolism and have been reported to be involved in carcinogenesis. As the organ that controls metabolism, the liver in particular shows the strong involvement of PPARs. It is not clear whether each subtype of PPARs works to promote or inhibit cancer. In this paper, we describe the associations between PPAR and HCC.

## 2. Relevance of PPAR**α** and the Pathogenesis of HCC

PPAR*α* expression has a major impact on the maintenance of mitochondrial beta-oxidation [15]. The ligand in the natural product of PPAR*α* assumes the form of a fatty acid, and fenofibrates that reduce triglycerides act as a PPAR*α* agonist [16]. It has been controversial whether it promotes or suppresses cancer growth. Several reports have described that it has an inhibitory effect on cancer [17–20]. PPAR*α* agonist suppressed the inhibition of angiogenesis via excess production of thrombospondin (TSP)-1. In addition, PPAR*α* acts as a master regulator of inflammation, showing an anti-inflammatory action in suppressing interleukin-1*β*, TNF-*α*, and ICAM-1 [21]. The antiangiogentic and anti-inflammatory effects promote the suppression of tumor growth by improving microenvironment.

On the other hand, hepatocellular carcinoma or hepatomegaly has been known to occur when PPAR*α* agonists were administered for a long time to mice or rats [22]. Peters et al. [23] employed fenofibrates in PPAR*α* knockout mice and wild-type mice to investigate the cell cycle regulatory proteins. The expression levels of cell cycle regulatory proteins did not change significantly between the knockout mice and wild-type mice in the steady state. On the other hand, the expressions of cyclin D1, cyclin E, cyclin-dependent kinase 2, CDK4, and proliferating cell nuclear antigen increased remarkably in wild-type mice that were administered fibrates. However, their expression levels did not change in the knockout mice. In this study, it was revealed that PPAR*α* was involved in the regulation of the cell cycle. In addition, HCV core transgenic mice showed a high rate of hepatocellular carcinoma from fatty liver and hepatomegaly [24]. In this model, by knocking down PPAR*α*, the development of hepatocellular carcinoma was suppressed [25]. In brief, HCV core, which can lead to carcinoma, is abnormally sustained by PPAR*α* activation. In this mouse model, the overexpression of several genes related to fat was observed. PPAR*α* leads to carcinoma from fatty liver through these genes (fatty acid translocase (FAT) and fatty acid transport protein (FATP)). Appropriate stimulation of PPAR*α* suppresses the cancer through the microcirculation. On the other hand, continuous abnormal stimulation promotes the cancer.

## 3. Relevance of PPAR**γ** and the Pathogenesis of HCC

PPAR*γ* expression is observed in adipose cells and macrophages. Furthermore, PPAR*γ* is expressed in the liver in a hypernutrition state such as fatty liver [26]. The expression of PPAR*γ* varies in hepatocellular carcinoma and is reported to be at the same level [27], a higher level [28], or a lower level [29] in comparison with normal liver. It has been reported that PPAR*γ* inhibits hepatocellular carcinoma [28, 30, 31] and other carcinomas [32–35] in many *vitro* studies. These control epithelial-mesenchymal transition (EMT) and prevent the invasion and metastasis of carcinoma. The overexpression of PPAR*γ* inhibits the metastasis of carcinoma by increasing E-cadherin through TIMP3 [36]. PPAR*γ* has been also revealed to be involved in cell cycle arrest [36]. These mechanisms have been reported to act through p21 and p53 [37]. Additionally, the pathway of p27 has been reported to be independent [29]. Furthermore, PPAR*γ* induces apoptosis directly through Fas, resulting in an inhibitory effect on carcinoma [31].

## 4. PPAR**δ** and HCC

 PPAR*δ* is expressed universally. PPAR*δ* plays an important role in lipid and glucose metabolism and has been implicated in obesity-related metabolic disease. The involvement of PPAR*δ* in colon cancer has been reported. It is reported that PPAR*δ* in the cells indicates extreme malignancy in a colon cancer cell [38]. PPAR*δ* is a gene derived from TCF/*β*-catenin pathway. However, there are few reports on the association between PPAR*δ* and HCC. EpCAM is a useful cancer stem cell marker in HCC. Activation of the Wnt/*β*-catenin signaling in the nucleus causes it to migrate along with *β*-catenin, FHL2, and intracellular domain (EpICD) of EpCAM [39]. The association between PPAR*δ* and HCC through Wnt/*β*-catenin signaling should be clarified in the future.

## 5. PPARs and Cancer Stem Cell

The cancer stem cell theory has been proposed in recent years to be applicable to many types of cancer [40–43]. In this theory, there are low-frequency cancer cells that have the potential for self-renewal, pluripotency, tumorigenicity, and asymmetric division like bone marrow stem cells and progenitor cells in normal tissue. The cancer stem cell theory itself was first suggested in the 1970s [44], and since then, it has been difficult to be confirmed experimentally. However, it was reported that there is a high tumorigenic fraction, CD34^+^CD38^−^, in human acute myeloid leukemia in 1997 [40]. The presence of cancer stem cells was subsequently reported in various types of cancer. Now, this concept has been established by many studies. PPAR is involved in the control of cancer because it acts in the control of the cell cycle. Therefore, it seems that some link exists between PPAR and cancer stem cells. However, there are still few reports concerning cancer stem cells [45]. Chearwae and Bright [46] reported that PPAR*γ* agonists inhibited growth and expression of brain tumor stem cells by inhibition of EGF/bFGF signaling through Tyk2-Stat3 pathway and the expression of PPAR*γ*. It is also notable that there is a relationship between cancer stem cells and EMT. Mani et al. [47] reported that EMT-generating cells had the properties of stem cells. If PPAR*γ* is involved in EMT, it is possible that PPAR has a role in the mechanism of metastasis of the cancer stem cells. PPAR*γ* agonists might regulate cancer stem cells. However, cancer stem cells have reduced the expression of PPAR*γ*, allowing cells to escape from the control of the normal cell cycle [45].

## 6. PPARs and Treatment

PPARs may become the target of cancer treatment. Particularly, PPAR has an important role in carcinogenesis from fatty liver cells. It is expected that the thiazolidinediones (TZDs), which act on PPAR*γ*, can be used to treat hepatocellular carcinoma. TZDs are expected to inhibit the proliferation of hepatocellular carcinoma. Troglitazone was the first TZD to become clinically available. However, troglitazone produced liver damage at a high rate [48] and therefore can no longer be used. Today, we can only use pioglitazone in Japan. Pioglitazone is reported to have a certain therapeutic effect for NASH. Although it has been widely used in clinical practice, sufficient effectiveness has not been reported fore hepatocellular carcinoma. However, aggressive usage has become more difficult, and some reports suggested that pioglitazone caused weight gain and edema as a side effect, and also bladder cancer [49]. However, PPAR*γ* activity exists in other drugs and natural products. For example, telmisartan [50], a kind of angiotensin II receptor blockers that has some PPAR*γ* activity, and side effects such as weight gain do not occur. Chearwae and Bright [46] reported PPAR*γ* agonist, and all-*trans* retinoic acid combination therapy had strong inhibitory effects on brain tumor stem cells. All-*trans* retinoic acid is used for acute promyelocytic leukemia (APL). Such use of retinoic acid is a differentiation induction therapy used only in a cancer. APL is caused by abnormal molecules called PML/RAR*α* made by translocation t(15; 17).

With PPAR agonists alone, it is difficult to treat cancer. However, there is a possibility for its use in the treatment of HCC in the future, such as in combination with molecular target drugs like retinoic acid. Therefore, curative effects for HCC are expected in the future.

## 7. Conclusion

PPARs play an important role in the generation of fatty liver. *Abnormal* stimulation of PPAR*α* generates HCC through fatty liver. Particularly, infection of HCV causes abnormal stimulation of PPAR*α*. In HCC, it is not clear whether PPAR*γ* promotes cancer or can control it. At present, PPAR*γ* suppresses cancer *in vitro*. Some reports describe that PPAR*γ* affects control of cancer stem cells. In cancer stem cell theory, it is thought that cancer stem cells participate in chemoresistance and recurrence. Accordinly, it is important to induce cancer stem cells to become noncancer stem cells (mature cancer cells). Nuclear receptor agonists like PPARs might be the key for differentiation therapy. PPARs might be useful to target cancer stem cells in inducing the differentiation of HCC, because the expression of PPARs has been implicated in the regulation of cell cycle of hepatocytes and adipocytes in the liver.
